# A rare novel mutation in *TECTA* causes autosomal dominant nonsyndromic hearing loss in a Mongolian family

**DOI:** 10.1186/1471-2350-15-34

**Published:** 2014-03-19

**Authors:** Haihua Bai, Xukui Yang, Temuribagen, Guilan, Suyalatu, Narisu Narisu, Huiguang Wu, Yujie Chen, Yangjian Liu, Qizhu Wu

**Affiliations:** 1 Inner Mongolia University for the Nationalities, Tongliao, Inner 028000, Mongolia; 2 BGI-Shenzhen, Shenzhen, Guangdong 518083, China; 3 Genome Technology Branch, National Human Genome Research Institute, National Institutes of Health, Bethesda, MD, USA; 4 Department of Developmental Biology, Washington University School of Medicine, St. Louis, MO 63110, USA

**Keywords:** *TECTA* gene, Mongolian family, Autosomal dominant nonsyndromic hearing loss

## Abstract

**Background:**

The genetic basis of autosomal dominant nonsyndromic hearing loss is complex. Genetic factors are responsible for approximately 50% of cases with congenital hearing loss. However, no previous studies have documented the clinical phenotype and genetic basis of autosomal dominant nonsyndromic hearing loss in Mongolians.

**Methods:**

In this study, we performed exon capture sequencing of a Mongolian family with hereditary hearing loss and identified a novel mutation in *TECTA* gene, which encodes α -tectorin, a major component of the inner ear extracellular matrix that contacts the specialized sensory hair cells.

**Results:**

The novel G → T missense mutation at nucleotide 6016 results in a substitution of amino acid aspartate at 2006 with tyrosine (Asp2006Tyr) in a highly conserved zona pellucida (ZP) domain of α-tectorin. The mutation is not found in control subjects from the same family with normal hearing and a genotype-phenotype correlation is observed.

**Conclusion:**

A novel missense mutation c.6016 G > T (p.Asp2006Tyr) of *TECTA* gene is a characteristic *TECTA*-related mutation which causes autosomal dominant nonsyndromic hearing loss. Our result indicated that mutation in *TECTA* gene is responsible for the hearing loss in this Mongolian family.

## Background

Hearing loss is one of the most common sensory disorders in humans. Genetic factors are responsible for approximately 50% of cases with congenital hearing loss [1,2]. While 30% of hereditary hearing loss are syndromic associated with specific signs and medical problems, the other 70% are nonsyndromic [3]. The heredity of nonsyndromic hearing loss has recessive and dominant modes of inheritance. It is estimated that 80% of genetic forms of hearing loss is autosomal recessive and the remaining 20% is autosomal dominant [2]. Autosomal dominant nonsyndromic hearing loss (ADNSHL) is represented by heterogeneity of genetic and clinical features. To date, about 60 loci associated with ADNSHL have been mapped but only 24 genes have been identified [4].

As one of causative genes of ADNSHL, *TECTA* mutations have been identified in various types of hearing loss, age of onset, progression and frequency involvement in various populations [5-13]. This gene encodes α-tectorin, the major noncollagenous component of tectorial membrane which contacts the outer cochlear hair cells and has an important role in intracochlear sound transmission [14]. The α-tectorin is composed of three distinct modules: the entactin G1 domain, the zonadhesin (ZA) domain and the zona pellucida (ZP) domain [14]. Missense and nonsense mutations in the entactin G1 domain and ZP domain are associated with hearing impairment at mid-frequency, whereas mutations in the ZA domain primarily affect hearing at the high frequencies [13,15]. Phenotypes of hearing loss associated with *TECTA* can range from mild to severe and have pre or postlingual onset [13].

In this study, we examined the genetic basis of ADSHNL in a Mongolian family with hereditary hearing loss by exon-capture sequencing of known genes associated with hearing loss and identified a novel mutation in *TECTA* gene. This novel mutation causes the aspartate to tyrosine substitution (Asp2006Tyr) in the highly conserved ZP domain of α-tectorin. We further studied the genotype-phenotype correlation of mutation in *TECTA* gene with control subjects from the same family with normal hearing and confirmed that mutation in *TECTA* gene is responsible for the hearing loss in this Mongolian family.

## Methods

### Subjects

Patients of a Mongolian family suffered severe hearing impairment were diagnosed in the Affiliated Hospital of Inner Mongolia University for the Nationalities. Clinical examination showed that none of these patients has any other associated neurological signs, visual dysfunction or diabetes mellitus. Nineteen members of this Mongolian family participated in the study, of which fourteen showed clinical symptoms of hearing impairment. The study protocol was approved by the Committee on Clinical Investigation at the Inner Mongolia University for the Nationalities University. Informed written consent was obtained from all study participants and the study was in accordance with regulations by the Inner Mongolia University for the Nationalities University.

### Audiometric evaluation

Hearing impairment was evaluated by pure tone audiometry (PTA) in a sound-controlled room at frequencies ranging from 500 to 8000 Hz, according to standard protocols. The severity of hearing loss was defined as follows based on the mean PTA using the thresholds measured at 500, 1000, 2000, and 3000 Hz: normal hearing, below 20 dB; mild hearing impairment, 21 - 40 dB; moderate hearing impairment, 41 - 70 dB; severe hearing impairment, 71 – 95 dB; and profound hearing impairment, > 95 dB [16]. The range of hearing loss was described as follows depending on the PTA: low frequency, 125 - 1000 Hz; mid-frequency, 1000 - 4000 Hz; and high frequency, 4000 - 8000 Hz.

### Target exon capture sequencing

Approximately 4 mL of peripheral blood were collected from each study participant and genomic DNA was extracted using the QIAamp DNA Blood Mini Kit (Qiagen, Hilden, Germany). The qualified genomic DNA sample of the affected subject III_21_ was randomly fragmented by Covaris (Covaris S2, Massachusetts, USA) into segments ranging from 200 bp to 300 bp. These DNA fragments were purified and quantified, and then adapters were ligated to both ends. After amplified by ligation-mediated PCR (LM PCR), adapter-DNA fragments were hybridized to the capture-array of BGI (BGI, Shenzhen, China) to capture all target genes for 72h. After washing off non-hybridized fragments, captured library which contains the targeted exon sequences of 54 known genes associated with hearing loss was loaded on the Hiseq2000 platform (Illumina, San Diego, USA). Raw image files were processed by Illumina base calling Software 1.7 for base calling with default parameters and the sequences were generated as 90bp paired-end reads.

After removing adapter sequences and low quality sequence reads from the raw data, the remaining clean reads were mapped and aligned to the human reference genome (NCBI build 37.1/hg19) using Burrows Wheeler Aligner (BWA). SNPs and indels (insertion and deletion) were called by SOAPsnp and GATK (http://www.broadinstitute.org/gsa/wiki/index.php/, The Genome Analysis Toolkit), respectively. All variants of the samples were filtered by databases including dbSNP137 (http://hgdownload.cse.ucsc.edu/goldenPath/hg19/database/snp137.txt.gz), HapMap project (ftp://ftp.ncbi.nlm.nih.gov/hapmap), 1000 Genome Project (ftp://ftp.1000genomes.ebi.ac.uk/vol1/ftp), and an in-house database of BGI. After filtering, variants were further analyzed by SIFT and PolyPhen to predict the effect of mutation on the function of encoding proteins. Only variants that had significant impact on the function of the target protein were further analyzed.

### Sanger sequencing

Candidate variants observed via target exon capture sequencing were screened in other family members who participated the study using conventional capillary sequencing. Briefly, DNA fragments containing variants were PCR amplified from 10-40 ng of genomic DNA using a touch down protocol with primers listed in Table 1. PCR products were visualized on agarose gels, purified with Sephadex columns in accordance with the manufacturers’ protocols. Sequence analysis was performed with the Big Dye Terminator Cycle Sequencing Kit and the ABI PRISM 3730 DNA Analyzer (Applied Biosystems). Sequence traces were analyzed using the Sequencher 4.7 program (Gene Codes Corporation).

**Table 1:** **Primers of *TECTA *** **and *SLC26A4 *** **gene**

**Loci**	**Primer sequence (5'-3')**	**Size (bp)**
TECTA-F	GGTCACTTTCAAATGTAAAGG	443
TECTA-R	CACTGTCCCATCAAAGATGAC	
SLC26A4-F	GCTGATATCATGGTTTTTCATG	581
SLC26A4-R	ACACAAATAGGACTATTGAAGG	

## Results

### ADSHNL diagnosis and clinical study

**Figure 1:**
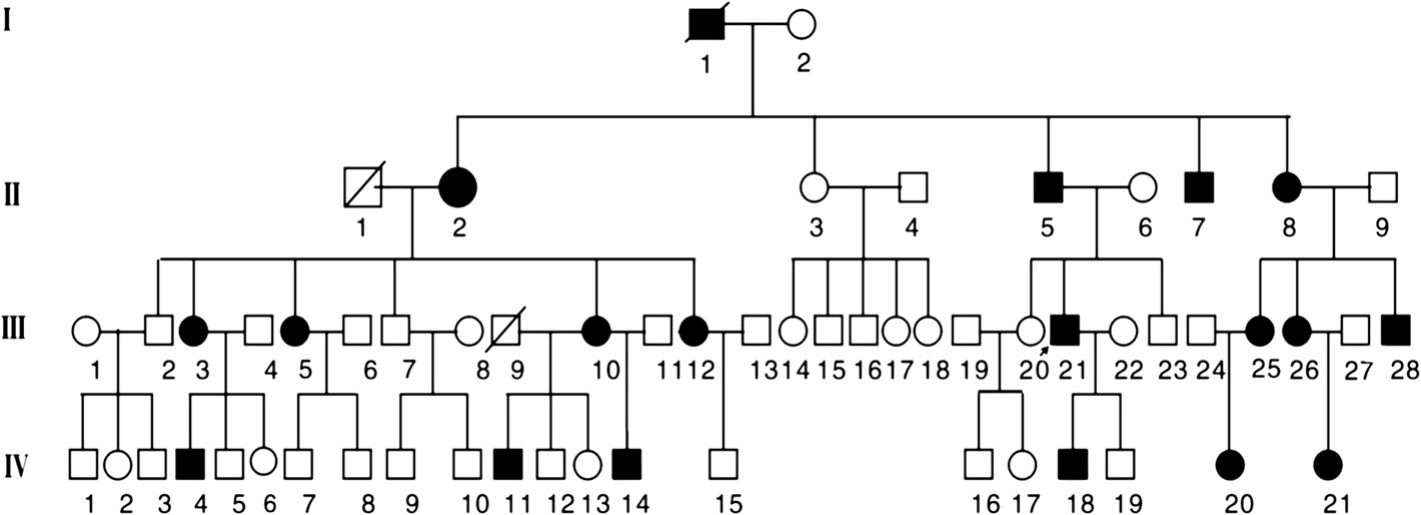
**Pedigree of the Mongolian family.** Black and white symbols indicate the affected and the unaffected subjects, respectively.

**Figure 2:**
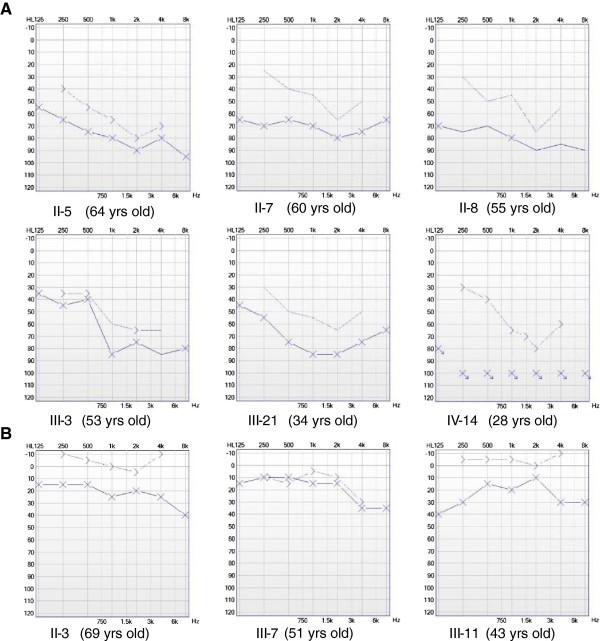
**Pure tone audiometry recording of representative members in the Mongolian family. A**. audiograms of six different patients in three generations (only audiograms of left ear is shown). X: frenquency; Y: intensity (dB). **B**. audiograms of the left ear of three different control subjects in two generations. The age of each subject at the time of tested was indicated.

We ascertained a Mongolian family with autosomal dominant nonsyndromic hearing loss. The pedigree comprises four generations with 19 affected family members (9 males and 10 females), 14 of whom are willing to participate in this study (Figure 1). There was no evidence of any other cause of hearing impairment, except for one patient who had undergone stapes replacing surgery for unilateral otosclerosis. This patient was included in this study but only audiometric data from her other non-surgical ear were used. The first symptoms of hearing impairment were self-reported at ages ranging from 1 to 69 years. No vestibular symptoms were reported. Otoscopy was normal in all subjects except the above-mentioned patient. Pure tone audiograms (PTA) were symmetric and often displayed a so-called cookie-bite shape, which indicates that the survey frequencies are predominantly affected (Figure 2). The highest threshold was most often found at 1000 Hz and followed by 2000 Hz. The PTA was usually within the range of 40–120 dB and apparently independent of age. By contrast, the PTA from control subjects with normal hearing was in the range of 0–40 dB (Figure 2).

### Identification of candidate mutation

To understand the genetic basis underlying the hearing loss in this Mongolian family, we took the candidate gene approach to screen 54 known hearing loss associated genes with exon capture followed by deep sequencing of the DNA sample from the patient III21. We then analyzed the SNPs and indels mutation from sequencing reads. The bioinformatic analysis identified four missense variants (p.Arg371Gly, p.Val932Ala, p.Ser1724Asn and p.Asp2006Tyr) and four synonymous variants (p.Ala495Ala, p.Ile752Ile, p.Tyr935Tyr, p.Ser1878Ser) in coding region of *TECTA* (Table 2), which encodes α-tectorin, a major component of the inner ear extracellular matrix that contacts the specialized sensory hair cells. One missense mutation (p.Cys282Gly) in *SLC26A4* was also called in the aforementioned analyzed strategy. No other mutation was identified in the rest of known hearing loss associated genes, including *ACTG1*, *CCDC50*, *COCH*, *COL11A2*, *DFNA5*, *DIAPH1*, and *EYA4 et al.* for this patient sample.

**Table 2:** Mutations identified by exon-capture sequencing

**Variants**	**Rs_ID**	**MAF-dbSNP**	**MAF-HapMap**	**MAF-1K project**	**MAF-in-house**
p.Arg371Gly	rs612969	0.5	0.394	0.4432	0.3892
p.Ala495Ala	rs536069	0.629	0.533	0.6007	0.4627
p.Ile752Ile	rs10502247	0.449	0.474	0.402	0.4988
p.Val932Ala	rs520805	-	0.387	0.3526	0.3843
p.Tyr935Tyr	rs586473	0.588	0.504	0.5696	0.4663
p.Ser1724Asn	rs526433	-	0.993	0.9936	0.9964
p.Ser1878Ser	rs2155369	-	0.314	0.2244	0.4181
p.Asp2006Tyr	novel	-	-	0	0

**Figure 3:**
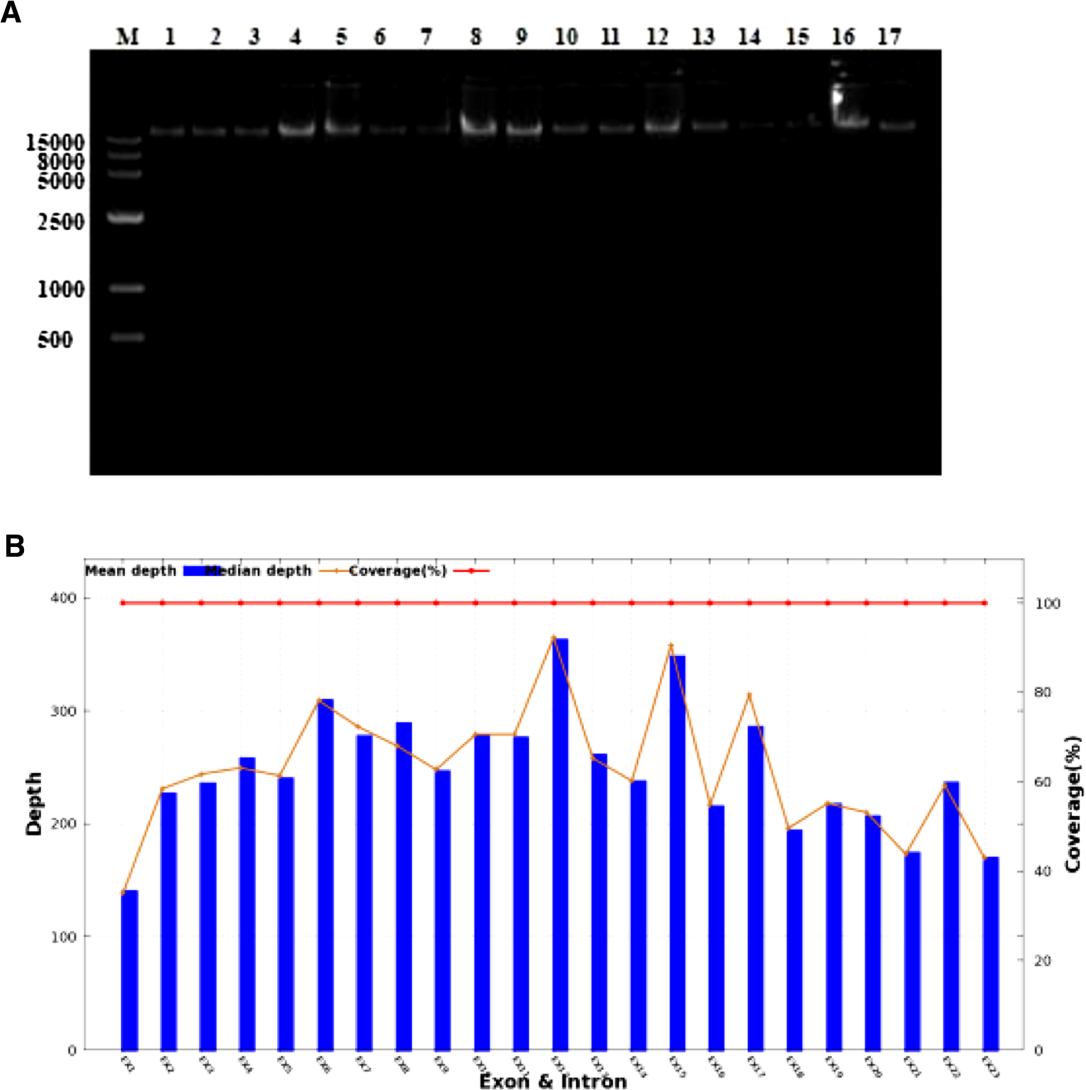
**Genomic DNA extraction and TECTA exon-capture sequencing report. A**. Agarose gel examination of genomic DNA quality. 1:I2; 2:II3; 3:III15; 4: III7;5: IV6; 6: II2;7: II7;8: II5;9: II8;10: III3; 11: III10;12: III21;13: III26;14: III28;15: IV4;16: IV14; 17:IV18. **B**. *TECTA* exon-capture sequencing report. Mean depth(X): the sequencing depth of each exon. Median depth(X): This number of depth is the median depth when depth of all sites was in an ascending sort. Coverage (%): Divide the total length of the target regions by bases generated by sequencing.

The sequencing coverage of every exon of *TECTA* was up to 99%, and the depth of each exon was close to the average median-depth across 23 exons, indicating sequencing in our study is deep enough for identifying all possible variants (Figure 3B). This suggests the above-mentioned variants are the possible causative mutations impairing the hearing of this Mongolian family. To narrow down the candidate variants of *TECTA*, we then filtered these variants with four databases (dbSNP, HapMap, 1000 project and in-house database of BGI) with a minor allele frequency (MAF) of 0.005. Only p.Asp2006Tyr was left after filtering, suggesting a novel variant in *TECTA*.

### Genotype-phenotype correlation study

The *TECTA* gene is known as a causative gene for DFNA8/DFNA12 and DFNB21 hearing loss in humans, and *SLC26A4* is related to autosomal recessive hearing loss. The missense mutations p.Asp2006Tyr of α-tectorin and p.Cys282Gly of SLC26A4 are heterozygous, which fits the autosomal dominant inheritance model. These two mutations have not been reported previously based on the public databases. The p.Asp2006Tyr of α-tectorin and p.Cys282Gly of SLC26A4 were predicted to be pathogenic mutations by SIFT and PolyPhen. Thus, p.Asp2006Tyr of α-tectorin and p.Cys282Gly of SLC26A4 were considered as candidate causative mutations of hearing loss in the studied Mongolian family. Therefore, we further screened these mutations in other affected subjects using Sanger sequencing for a genotype phenotype correlation analysis with primers listed in Table 1.

**Figure 4:**
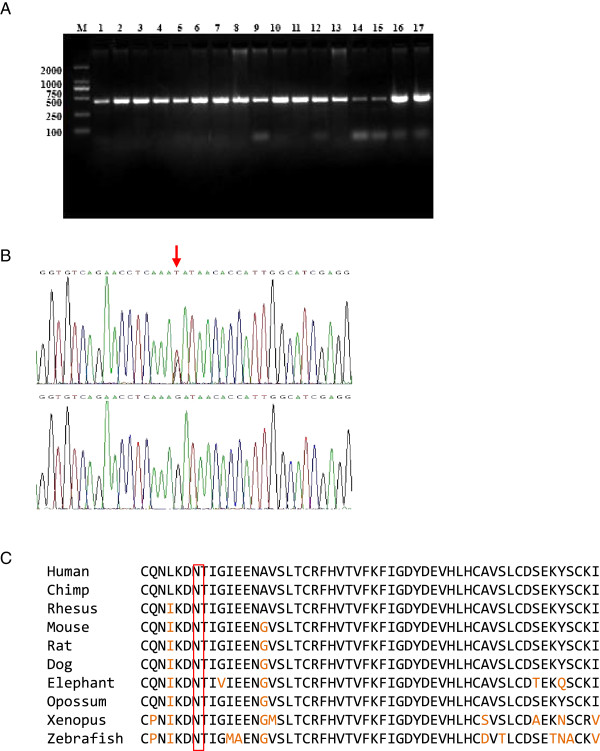
**Sequencing analysis of novel missense mutation in exon 20 of *TECTA*. A**. Agarose gel analysis of PCR product *TECTA* exon 20. 1:I2; 2:II3; 3:III15; 4: III7;5: IV6; 6: II2;7: II7;8: II5;9: II8;10: III3; 11: III10;12: III21;13: III26;14: III28;15: IV4;16: IV14; 17:IV18. **B**. Representative Sanger sequencing trace showing heterozygous missense mutation at G6016T (arrow) from patients and control subjects. **C**. Multi-species alignment of amino acids encoded by exon 20 of *TECTA* across different vertebrates. The conserved aspartate residue is highlighted in red.

Sequencing of three patients and three control subjects in the family showed that c.844 T > G (p.Cys282Gly) in *SLC26A4* is also present in controls, ruling out the association of p.Cys282Gly in *SLC26A4* with hearing loss in the studied Mongolian family. We then confirmed the presence of the novel missense mutations c.6016 G > T (p.Asp2006Tyr) of the *TECTA* gene in exon 20 of the rest of all 13 participated subjects with hereditary hearing loss (Figure 4B). The affected members in this family (II-2, III-3, III-5, and III-10) were heterozygous for the mutation, but not detected in the II-1. This is consistent with a complete segregation of the variant allele with the phenotype status in this family (Figures 1 and 4B). This mutation is located in the ZP domain of α-tectorin which is highly conserved across species (Figure 4C). We further sequenced DNAs of all the normal control subjects of the family, plus screened additional 201 healthy individuals from a random Mongolian population sample that we have deep sequencing data. We did not find the presence of the above-mentioned p.Asp2006Tyr mutation in the *TECTA* gene (data not shown), demonstrating the genotype-phenotype correlation of the mutation in *TECTA* gene with the hearing loss in the Mongolian family.

## Discussion

*TECTA* encodes α-tectorin, the major component of extracellular matrix contacting cochlear hair cells and transmitting sounds [14]. Mutations in α-tectorin have been detected in ADNSHL in various populations across different ages of onset and progressions [5-13], accounting for ~4% of all cases [13]. Interestingly, previous studies showed that mutations affecting three different domains (entactin G1 domain, ZA domain, and ZP) present clinically different phenotypes. Missense mutations on the ZA domain are normally affecting hearing at the high frequencies, whereas mutations on entactin G1 domain and ZP domain are linked to mid-frequency hearing impairment [13,15]. ADNSHL patients with mutations in α-tectorin display various severity and onset of hearing loss [13].

In this study, we did exon-capture followed by next-generation sequencing to screen 54 known hearing-loss associated genes for mutations in a Mongolian family with hereditary hearing loss. We identified four missense variants (p.Arg371Gly, p.Val932Ala, p.Ser1724Asn and p.Asp2006Tyr) and four synonymous variants (p.Ala495Ala, p.Ile752Ile, p.Tyr935Tyr, p.Ser1878Ser) in coding regions of *TECTA*. Among these, a novel variant, p.Asp2006Tyr was found to be implicated as the pathogenic missense mutation causing hearing loss. All *TECTA* mutations described to date have shown significant genotype-phenotype correlation with hearing loss [5-13]. In our study, the novel heterozygous mutation p.Asp2006Tyr on *TECTA* is only present in affected members of the family and is not found in any of the control subjects in the family plus a fairly large population sample of the same ethic group. Consistent to previous reports that mutations on the ZP domains are associated with mid-frequency hearing loss, the hearing of affected subjects was predominantly affected at the frequency between 1000 Hz and 2000 Hz. The clinical phenotype of hearing loss in this family displays early onset (as early as 1 year old). However, no correlation between age and severity of hearing impairment has been observed.

Several recently developed mouse lines with human mutations on Tecta provide extremely useful information for understanding the mechanism of hearing loss in the affected families [17-19]. Mice with missense mutation in the ZP domain of Tecta (Tecta^Y1870C/+^, Tecta^L1820F,G18724D/+^ and Tecta^C1837G/+^) display hearing loss in mild-frequency range, phenocopying the clinical observation with affected patients [17,19]. By contrast, the hearing impairment in mice carrying missense mutation in the ZA domain of Tecta (Tecta^C1509G/+^ and Tecta^C1619S/+^) is more subtle and slightly different from what had been reported in patients [18,19]. While the Tecta^C1509G/+^ mouse had increased reverse transduction [18], the Tecta^C1619S/+^ mouse was surprisingly found to have a stable rather than progressive deterioration hearing phenotype [19]. The authors suggested the genetic traits of the mouse lines could be a possible explanation for the different deafness phenotype in mouse and human caused by the same mutation. Likewise, the age-independency of hearing loss in the large Mongolian family under this study is probably dues to the slight difference in their individual genetic background. A knockin mouse line with Tecta^D1619Y/+^ will be very useful to investigate how the point mutation identified in current study affects the onset of deafness under various mouse genetic backgrounds.

This novel variation p.Asp2006Tyr in the ZP domain was a substitution of aspartate with a hydrophilic side chain to tyrosine with a hydrophilic-neutral chain. Previously, various mutations in the ZP domain (C1837G, R2021H, and Y1870C) of *TECTA* had been shown within the cytoplasm, suggesting the defect for the mutants to be secreted outside of the cell properly [20]. It seems likely that the p.Asp2006Tyr mutation in the ZP domain from this study might also cause similar defects in the secretion of α -tectorin. However, it is of note that the secretion defect of mutations in the ZP domain was drawn from truncated constructs of *TECTA* without N-terminal secretion peptide signal. Further cellular studies with full-length constructs are necessary to elucidate the exact mechanism of α-tectorin’s role in ADNSHL.

## Conclusion

This study documents a novel missense mutations c.6016 G > T (p.Asp2006Tyr) of the *TECTA* gene that causes autosomal dominant nonsyndromic hearing loss in a Mongolian family. This expands the mutation spectrum of *TECTA* gene in nonsyndromic hearing loss. Our study also exemplifies the application of deep sequencing in identifying disease causative mutations from a pedigree.

## Competing interests

The authors declare that they have no competing interests with respect to the present article.

## Authors’ contributions

HB designed the study and family recruitment, performed experiments, analyzed and interpreted the data, drafted the manuscript and obtained funding. QW designed the study, conducted clinical diagnoses and obtained funding. XY selected and designed the targets capture, performed experiments, analyzed and interpreted the data. T conceived of the study concept, drafted the manuscript, and analyzed and interpreted the data. NN supervised the study design. YL analyzed and interpreted the data. All other authors provided technical assistance, and all authors read and approved the final manuscript.

## Pre-publication history

The pre-publication history for this paper can be accessed here:

http://www.biomedcentral.com/1471-2350/15/34/prepub
